# Association between resilience and a functional polymorphism in the serotonin transporter (SLC6A4) gene: A meta-analysis

**DOI:** 10.17179/excli2020-2660

**Published:** 2020-08-19

**Authors:** Yeimy González-Giraldo, Diego A. Forero

**Affiliations:** 1 Departamento de Nutrición y Bioquímica, Facultad de Ciencias, Pontificia Universidad Javeriana, Bogotá, Colombia; 2 Center for Psychosocial Studies for Latin America and the Caribbean, Universidad Antonio Nariño, Bogotá, Colombia; 3 School of Health and Sports Sciences, Fundación Universitaria de Área Andina, Bogotá, Colombia

**Keywords:** resilience, genetics, SLC6A4 gene, 5HTTLPR polymorphism, meta-analysis

## Abstract

Resilience is a mechanism used by humans to adapt to adverse situations. It is a protective factor against mental health problems. This process can be influenced by environmental and genetic factors. Several genes have been associated with interindividual differences in resilience levels, but the results are inconclusive. Therefore, the aim of this meta-analysis was to evaluate the effect of a functional polymorphism (5-HTTLPR) in the* SLC6A4* gene on resilience levels. A search in PubMed, HugeNavigator and Google Scholar databases was carried out and 16 studies about the association of 5-HTTLPR polymorphism and resilience in humans were identified. The OpenMeta[Analyst] program was employed to perform statistical analysis using a random-effects model. The final analysis included 9 studies, for a total of 4,080 subjects. Significant results were found when the standardized mean differences (SMD) of *LL* and *SL* carriers were compared, (SMD: -0.087 (confidence interval: -0.166 to -0.008; *I^2^: *0 %); *P* value: 0.031). A significant result was also found in an analysis comparing *SS/SL* versus *LL* genotypes (SMD: -0.231; confidence interval: -0.400 to -0.061, *P* value: 0.008; *I^2^: *0 %). This is the first meta-analysis performed to identify the pooled association of a functional polymorphism in the serotonin transporter gene and resilience. The current results suggest that the *L/L* genotype is associated with resilience. Further studies are necessary to elucidate the role of genetics on the resilience mechanisms.

## Introduction

Resilience is a mechanism used by humans to adapt to and respond positively to stressful events that can occur during any time in life (Wu et al., 2013[48]). This process works as a protective factor against mental health problems. Moreover, it is a factor that has important implications for several aspects, such as the development of clinical complications and response to the treatment, for a large number of several chronic diseases (Kim et al., 2019[27]), in addition to psychiatric disorders. Different studies have demonstrated that lower levels of resilience are associated with depression (Kermott et al., 2019[26]), post-traumatic stress disorder (Wrenn et al., 2011[47]), and represents a risk for coronary heart disease (Bergh et al., 2015[6]). In contrast, high levels of resilience have been associated with a better response to treatments or recovery for chronic diseases (Kim et al., 2019[27]; Wrenn et al., 2011[47]), as cancer (Seiler and Jenewein, 2019[39]). Moreover, this mechanism influences the fluctuations in pain in patients with rheumatoid arthritis (Strand et al., 2006[44]). These variations in the resilience levels in humans are influenced by biological factors (Feder et al., 2019[15]) and by environmental and psychosocial factors (Liu et al., 2018[30]). 

Genetic mechanisms are one of the biological factors affecting the resilience (Navrady et al., 2018[32]). Some investigations have determined that this process has a heritability of approximately 31 %, with sex differences (Amstadter et al., 2014[3]). These results suggest that specific genes could be playing an important role in this psychological process. A genome-wide association study (GWAS) has identified that in European soldiers four Single Nucleotide Polymorphisms (SNPs) in *DCLK2 *gene are related to self-assessed resilience, in addition to variants in the *KLHL36 *gene (Stein et al., 2019[43]). On the other hand, studies focused in evaluating candidate genes have found that some polymorphisms in genes involved in synaptic plasticity, such as *SLC6A4, BDNF, DRD4* and *COMT,* have been associated with resilience levels in people with or without diseases (Carli et al., 2011[7]; Kang et al., 2013[25]; La Greca et al., 2013[28]; Niitsu et al., 2019[33]). In addition to SNPs, changes in expression of dopaminergic genes have also been correlated with differences in resilient responses (Azadmarzabadi et al., 2018[4]). 

Until now, the 5-HTTLPR (rs4795541) is one of the most studied polymorphisms in works about genetics of resilience mechanisms (Niitsu et al., 2019[33]). This polymorphism is located in the *SLC6A4* gene, which encodes the serotonin transporter. 5-HTTLPR has two alleles generated by a deletion/insertion of 44 base pairs; these alleles, short (*S*) and large (*L*), are constituted by 14 and 16 repeats, respectively (Heils et al., 1996[22]). *L* allele induces higher expression levels, whereas the *S* allele is associated with a lower expression and activity of the serotonin transporter (Lesch et al., 1996[29]). This functional polymorphism has been associated with major depressive disorder, stress, alcoholism, cognition, and other traits and diseases (Gatt et al., 2015[18]). However, several inconclusive and contradictory results have been reported regarding some traits and diseases (Culverhouse et al., 2018[12]; Smoller, 2016[41]). 

It is known that serotonin could modulate response to stress, and therefore, the availability of its transporter could be influencing mechanisms as resilience (Feder et al., 2009[16]). Mixed results have been observed between 5-HTTLPR and resilience, since both alleles, *S* and *L*, have been associated with differences in its levels. For example, in a previous study (Stein et al., 2019[43]), *S* carriers had a lower level of resilience. In contrast, in individuals with a high exposition to childhood traumas, the resilience levels were lower in *L* carriers (Carli et al., 2011[7]), and other studies have not found significant associations (O'Hara et al., 2012[34]). In this work, we aimed to perform a meta-analysis in order to clarify the effect of 5-HTTLPR on resilience levels in humans. 

## Methods

### Search strategy and inclusion criteria

**Figure 1:**
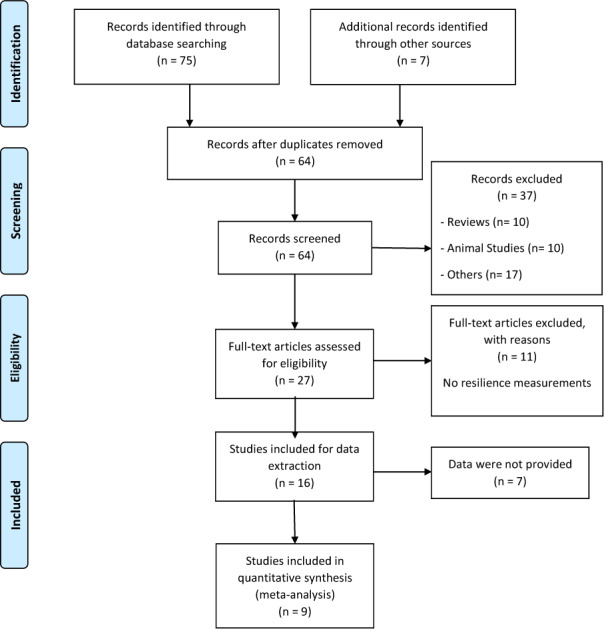
PRISMA flow diagram

We performed this meta-analysis following the PRISMA statement (Moher et al., 2009[31]) and previous recommendations (Forero et al., 2019[17]). The numbers of articles included and excluded are shown in Figure 1(Fig. 1). We did a search in different databases, such as PubMed, HugeNavigator and Google Scholar, to find original papers about resilience and the 5-HTTLPR polymorphism in humans, up to December 2019. Moreover, we searched in the reference lists of original and review articles to identify additional studies. We used the following terms for the search: “resilience”, “*SLC6A4*”, “5-HTTLPR”. The inclusion criteria were I) articles published in English language; II) articles that analyzed the association between 5-HTTLPR and resilience in humans; III) articles that reported the scores for resilience levels according to the three genotypes or alleles. Exclusion criteria were: I) studies that evaluated other polymorphisms in *SLC6A4* gene; II) studies that analyzed other measurements or scales; III) studies in animal models and review articles. 

### Data extraction and statistical analysis 

Once the studies were selected, the next step was to extract the following data from each article: Author and year, country, diseases/conditions, ethnicity, sample size, % males, mean age, genotyping methods, Hardy Weinberg Equilibrium, resilience scales and scores (mean and Standard Deviation -SD-) and genotypic and allelic frequencies (Tables 1(Tab. 1), 2(Tab. 2)). In case that these data were not available in the publications, the authors were contacted to ask for the missing information. Two authors independently performed this process. Finally, the analyses were conducted using the open-source and cross-platform software called OpenMeta[Analyst] (Wallace et al., 2009[46]), in which a Random-Effects Model was implemented, in order to calculate the effect size using the Standardized Mean Difference (SMD) metric. Herein, the means and standard deviations of resilience scores for each 5-HTTLPR genotype were used. Moreover, with this program, the *I^2^* statistic for heterogeneity analysis was calculated and forest plots were generated.

## Results

Following a systematic search in different databases, we identified 64 possible studies, of which 37 articles were excluded because they were reviews, animal studies or included other outcomes or scales. Sixteen articles were included for data extraction, however, information for 7 works was not found and the authors did not provide the data. Thus, the pooled statistical analysis reported in the present work was based on only nine studies (Figure 1(Fig. 1)), which included 4,080 subjects. The characteristics of these studies are presented in Tables 1(Tab. 1) and 2(Tab. 2) (References in Table 1 and 2: Carli et al., 2011[7]; Cicchetti and Rogosch, 2012[11]; Defrancesco et al., 2013[13]; Graham et al., 2013[20]; OʼHara et al., 2012[34]; Reinelt et al., 2015[35]; Sharpley et al., 2017[40]; Stein et al., 2009[42]; Taylor et al., 2014[45]). The mean ages were different between the publications and some works were carried out in patients with diseases such as cancer (Sharpley et al., 2017[40]), traumatic brain injury (Graham et al., 2013[20]) and bariatric surgery (Defrancesco et al., 2013[13]). Other studies were conducted in particular groups, such as normal children (Taylor et al., 2014[45]), older adults (O'Hara et al., 2012[34]), maltreated and no maltreated children (Cicchetti and Rogosch, 2012[11]), prisoners (Carli et al., 2011[7]) and healthy adult people (Reinelt et al., 2015[35]; Stein et al., 2009[42]). Genotypic frequencies and resilience scores are presented in Table 2(Tab. 2). 

In the included primary articles, the resilience levels were examined using different validated instruments, mainly the Connor-Davidson Resilience Scale (CDRISC) and the Resilience Scale (RS) (Table 1(Tab. 1)). In these instruments, higher scores represent higher resilience levels. Although the total scores in each particular scale are in different numerical ranges, the use of meta-analytical procedures based on Standardized Mean Differences (SMD) allowed their pooled comparison in this study.

**Figure 2:**
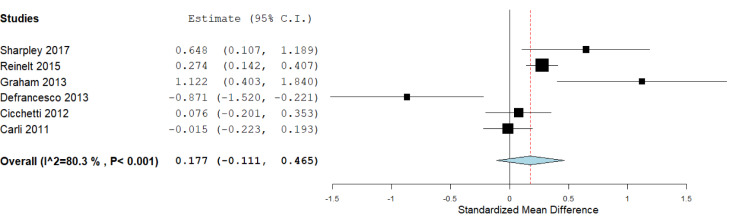
Forest plot for the meta-analysis of resilience scores in *S/S* and *L/L* carriers. *P *value: 0.228, a random-effects model

**Figure 3:**
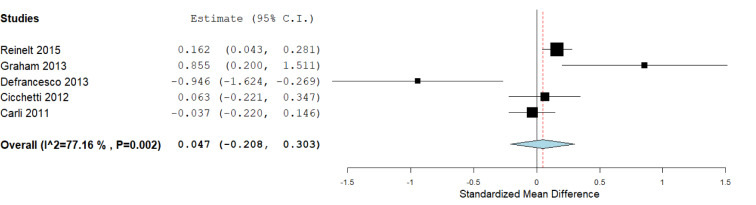
Forest plot for the meta-analysis of resilience scores in *S/S *and *S/L* carriers. *P *value: 0.715, a random-effects model

**Figure 4:**
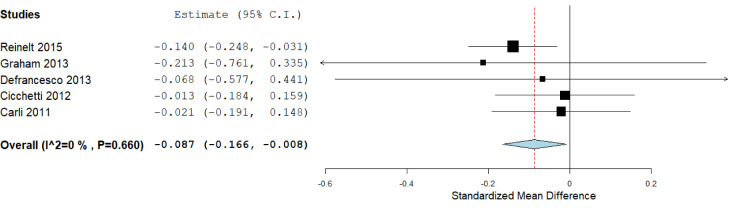
Forest plot for the meta-analysis of resilience scores in *L/L* versus *S/L* carriers. *P *value: 0.031, a random-effects model

According to the available data for each genotype, we performed the statistical analysis. First, we compared the carriers of *S/S *versus *L/L* genotypes (Figure 2(Fig. 2)), which included six studies, with 1,772 subjects. We did not find a significant association in this analysis: The standardized mean difference was 0.177 (confidence interval: -0.111 to 0.465; *P* value: 0.228). Heterogeneity between studies was observed: *I^^2^*: 80.3 % (*P* value: < 0.001). We also compared the *S/S* homozygotes with the *S/L* carriers (2,247 subjects), where we did not observe significant results (*P* value: 0.715), and the heterogeneity was of *I^^2^*: 77.16 % (*P *value: 0.002; Figure 3(Fig. 3)). When we compared the subjects carrying the *L/L* genotype with *S/L* carriers, a significant difference was observed, with a standardized mean difference of -0.087 (confidence interval: -0.16 to -0.008; *P *value: 0.031; Figure 4(Fig. 4)), and heterogeneity was not found (*I^^2^*: 0, *P* value: 0.66). In this case, five studies (1,671 subjects) were included.

**Figure 5:**
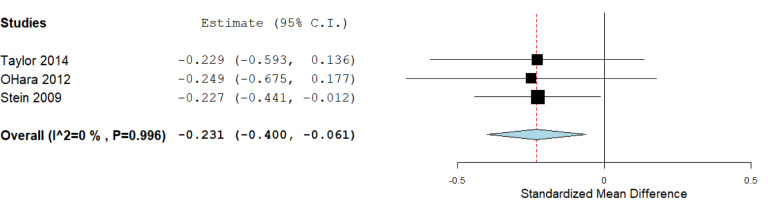
Forest plot for the meta-analysis of resilience scores in *S/S-S/L* versus *L/L *model, *P *value: 0.008. a random-effects model

For three studies (O'Hara et al., 2012[34]; Stein et al., 2009[42]; Taylor et al., 2014[45]), the means and SD for the three genotypes were not available, therefore, these works were included only in a genotypic analysis comparing *SS/SL* versus *L/L* carriers (Figure 5(Fig. 5)), which included 652 subjects. The standardized mean difference was -0.231 (confidence interval: -0.400 to -0.061; *P* value: 0.008). Heterogeneity between studies was not detected (*P* =0.99). 

Finally, we performed a leave-one-out meta-analysis in order to know whether the results were affected by a single study (Wallace et al., 2009[46]). The result for the *L/L* and *S/L* comparison resulted to be affected by excluding one study (Reinelt et al., 2015[35]), observing no significant results (Supplementary Figure 1). For the comparison between *S/S-S/L* versus *L/L*, the exclusion of one study (Stein et al., 2009[42]) affected the results (Supplementary Figure 2). In the analysis of *S/S* and *L/L*, we found that by excluding one study (Defrancesco et al., 2013[13]), the results became significant (Supplementary Figure 3): SMD 0.274 (confidence interval 0.027 to 0.521; *P* value: 0.030). 

## Discussion

Until now, some review articles have intended to describe previous works that studied candidate genes associated with resilience, suggesting that serotonergic, noradrenergic and dopaminergic systems play a pivotal role in this process (Niitsu et al., 2019[33]; Russo et al., 2012[37]). However, no study has performed a pooled statistical analysis to summarize the possible association between some polymorphisms in genes of these systems and resilience. Thus, we completed the first meta-analysis for the 5-HTTLPR polymorphism in the *SLC6A4* gene and the resilient responses. We observed statistically significant pooled differences between *L/L* and *S/L* carriers and between S/S-S/L versus L/L genotype groups on resilience levels. A sensitivity analysis found that these significant pooled results were affected by a single study.

Previous meta-analyses have suggested that *L* allele is a risk factor for some diseases, whereas *S* allele for others. These findings are related to the alteration of serotonin availability regulated by the transporter expression, which is modulated by the short and large alleles of 5-HTTLPR (Gatt et al., 2015[18]). This polymorphism has been widely studied in relation to depression, posttraumatic stress and bipolar disorder, suggesting an influence of the *S* allele (Smoller, 2016[41]). Additionally, these alleles also play a pivotal role in antidepressant treatment (Sahraian et al., 2013[38]). Due to mixed results observed in several studies, other authors suggest interpreting cautiously the findings, because the effect of this polymorphism is affected by other genetic variations and by the environment (Caspi et al., 2010[8]; Niitsu et al., 2019[33]).

A review article has suggested that the effects of 5-HTTLPR on resilience could be influenced by age, due to that in the adult population the *S/S* genotype could contribute to resilience, whereas in children the *L/L* genotype seems to be affecting the resilient responses (Niitsu et al., 2019[33]). However, in the current meta-analysis, we observed that by excluding a study that did not involve males in its analysis (Defrancesco et al., 2013[13]), the results became significant (Supplementary Figure 3). This allows us to suggest that the effect of 5-HTTLPR on resilient response could be influenced additionally by the gender. Previously, it has been reported that gender moderates the effect of these variations on several outcomes, for instance, affective disorders (Gressier et al., 2016[21]), and neuroticism (Chang et al., 2017[9]). It should be noted moreover that, resilience can be also affected by other psychological factors as the coping style (Hooberman et al., 2010[24]), which in turn is influenced by common genetic variants that also affect resilience, but in an opposite direction (Navrady et al., 2018[32]). 

Although a large fraction of publications have found that the *S/S* genotype and *S* allele affects negatively several outcomes, we observed that by comparing genotypes of 5-HTTLPR the results showed that *L/L* affects resilient responses, and only in a genotypic model comparison, the *S* allele was associated with resilience. These results should be interpreted with caution because of the fact that it was not possible to carry out several comparisons, due to the lack of availability of relevant data in the included articles. In addition, it was not possible to include several other studies in our analysis, taking into account that key data were missing on them (Agnafors et al., 2017[1]; Amstadter et al., 2012[2]; Beaver et al., 2011[5]; Delis et al., 2017[14]; Gibbons et al., 2012[19]; Hemmings et al., 2013[23]; Resnick et al., 2019[36]). Therefore, we suggest that all studies analyzing possible effects of genetic variations associated with resilience should report all scores and measures stratified by genotypes and alleles; this will improve further analyses. 

As we mentioned in the introduction, resilience is important for preventing mental illnesses, such as depression, anxiety, post-traumatic stress disorder, bipolar disorder, and it is an important factor during the treatments of chronic diseases. The World Health Organization has determined that approximately one in five people in post-conflict settings has a mental health problem (above mentioned) (Charlson et al., 2019[10]). Therefore, studying the genetic influences on this mechanism is quite important to elucidate the mechanisms underlying psychiatric disorders, especially in developing countries, where the burden of mental disorders is high.

## Acknowledgements

YGG was supported by a post-doctoral fellowship at Pontificia Universidad Javeriana. DAF was supported previously by grants from Colciencias.

## Conflict of interest

The authors declare no conflict of interest.

## Authors’ contributions

Both authors contributed to the study conception and design. Data collection and analysis were performed by Yeimy González-Giraldo, and Diego A. Forero. The first draft of the manuscript was written by Yeimy González-Giraldo and both authors commented on previous versions of the manuscript. Both authors read and approved the final manuscript.

## Supplementary Material

Supplementary material

## Figures and Tables

**Table 1:**
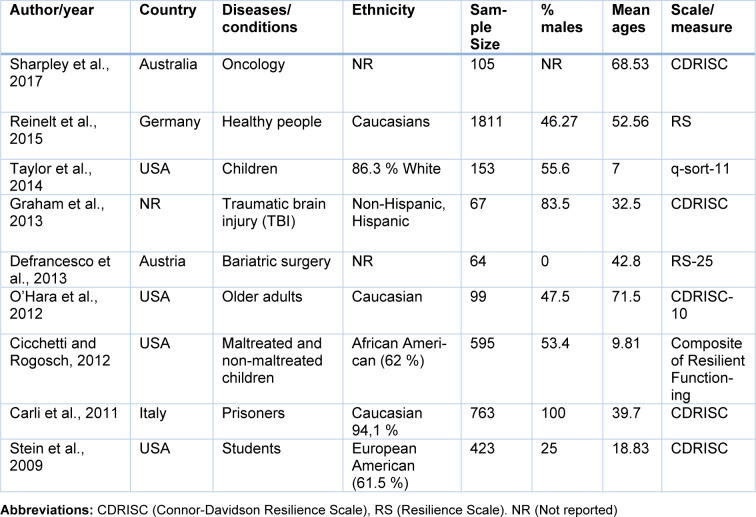
Characteristics of studies included in this meta-analysis

**Table 2:**
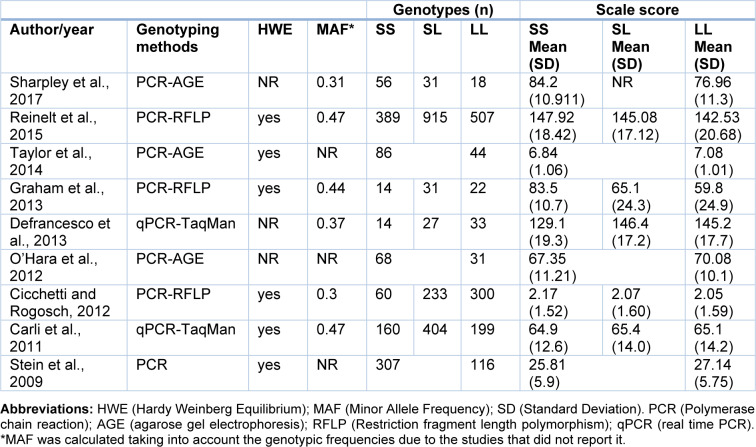
Distribution of genotypes and scores for resilience measurements
