# Supplementary material for: Association between resilience and a functional polymorphism in the serotonin transporter (SLC6A4) gene: A meta-analysis
Source: EXCLI J. 2020 Aug 19;19:1174–83. doi: 10.17179/excli2020-2660 (PMC7573178; doi:10.17179/excli2020-2660)
Supplement: Supplementary material [file EXCLI-19-1174-s-001.pdf]

EXCLI Journal 2020;19:1174-1183 – ISSN 1611-2156
Received: July 14, 2020, accepted: August 15, 2020, published: August 19, 2020

Supplementary material to:

ASSOCIATION BETWEEN RESILIENCE AND A FUNCTIONAL POLYMORPHISM IN THE SEROTONIN TRANSPORTER (*SLC6A4*) GENE: A META-ANALYSIS

Yeimy González-Giraldo, PhD[a,b]*, Diego A. Forero, MD, PhD[c]

[a] Departamento de Nutrición y Bioquímica, Facultad de Ciencias, Pontificia Universidad Javeriana, Bogotá, Colombia
[b] Center for Psychosocial Studies for Latin America and the Caribbean, Universidad Antonio Nariño, Bogotá, Colombia
[c] School of Health and Sports Sciences, Fundación Universitaria de Área Andina, Bogotá, Colombia

* **Corresponding author:** Dr. Yeimy González-Giraldo, PhD. Departamento de Nutrición y Bioquímica, Facultad de Ciencias, Pontificia Universidad Javeriana, Bogotá, Colombia. Telephone number: + 57 313 493 84 96, E-mail: yeimy.gonzalez@javeriana.edu.co

http://dx.doi.org/10.17179/excli2020-2660

This is an Open Access article distributed under the terms of the Creative Commons Attribution License (http://creativecommons.org/licenses/by/4.0/).

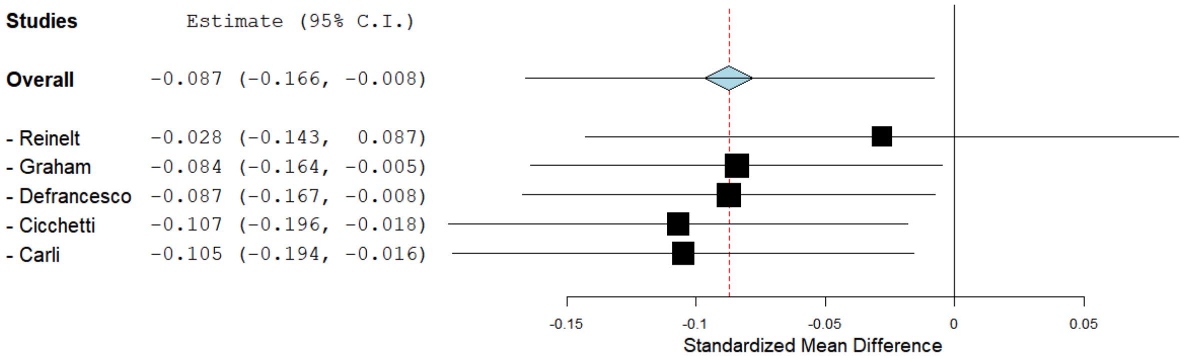

Supplementary Figure 1: Forest plot for Leave-one-out Meta-Analysis for resilience scores in *L/L* versus *S/L* carriers

EXCLI Journal 2020;19:1174-1183 – ISSN 1611-2156
Received: July 14, 2020, accepted: August 15, 2020, published: August 19, 2020

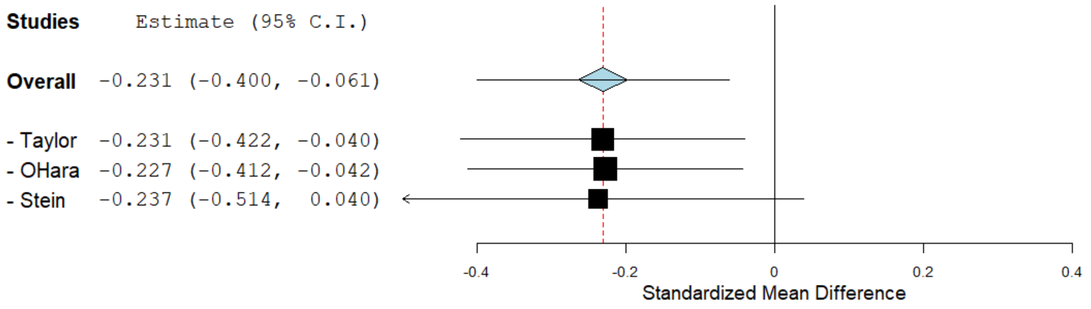

Supplementary Figure 2: Forest plot for Leave-one-out Meta-Analysis for resilience scores in *S/S-S/L* versus *L/L* carriers

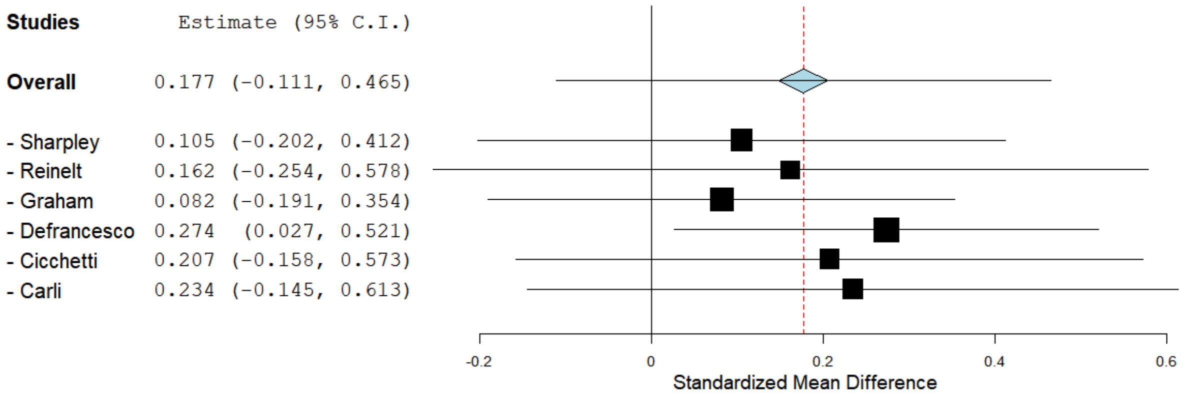

Supplementary Figure 3: Forest plot for Leave-one-out Meta-Analysis for resilience scores in *S/S* and *L/L* carriers
